# Auscultation of Bowel Sounds and Ultrasound of Peristalsis Are Neither Compartmentalized Nor Correlated

**DOI:** 10.7759/cureus.14982

**Published:** 2021-05-12

**Authors:** Anne Drake, Nicole Franklin, Jon W Schrock, Robert A Jones

**Affiliations:** 1 Emergency Medicine, Case Western Reserve University School of Medicine, Cleveland, USA; 2 Emergency Medicine, MetroHealth Medical Center, Cleveland, USA

**Keywords:** ultrasound, medical education, physical diagnosis

## Abstract

Objective

Auscultation of bowel sounds has been taught as a component of the physical examination since the beginning of the 20th century. However, there has been little research or consensus on the significance of listening in different quadrants. Some textbooks indicate that bowel sounds are the result of peristalsis in that region, while others state that bowel sounds can be generalized over the entire abdominal wall. With ultrasonography, peristalsis can be visualized in a dynamic and non-invasive manner. The purpose of this study was to determine the relationship between auscultation of bowel sounds and visualization of peristalsis with ultrasound, to understand whether or not bowel sounds and peristalsis are compartmentalized.

Methods* *

Study participants quietly lay supine, while one investigator positioned an ultrasound probe on the abdomen visualizing the small intestine, and a second investigator placed an EKO Digital Stethoscope (Eko Devices, Inc., Oakland, CA) directly adjacent to the probe auscultate for bowel sounds. During a two-minute interval, a third investigator noted every time a bowel sound was heard (A+), peristalsis was seen (U+), or a combined event (C+) occurred, recording the total number of events. Measurements were recorded from four quadrants (right upper quadrant {RUQ}, left upper quadrant {LUQ}, right lower quadrant {RLQ}, left lower quadrant {LLQ}) and the periumbilical region (PUR). Fisher Exact test was used to determine whether there were significant differences between the number of bowel sounds heard but not seen (A+) and those seen but not heard (U+) with sounds that were both seen and heard (C+). Significance was determined with p < 0.05.

Results

A total of 16 participants were included, with a combined 973 discrete bowel events, both auscultated and visualized. No quadrant showed a significant correlation between an isolated sound (A+) or peristalsis (U+) and a combined event (C+), indicating there were many events where an auscultated sound failed to correlate with observed peristalsis, and vice versa. The average p-value was 0.544, with a range of 0.052-1.00.

Conclusion

This study showed that there is no significant correlation between auscultated bowel sounds and peristalsis within a given region. This study calls into question whether auscultation of all four quadrants provides more meaningful information than auscultation of one central point of the abdomen.

## Introduction

The auscultative purpose of the stethoscope has allowed physicians a window into the body for well over a century [1,2]. Particularly, auscultation of bowel sounds has been a component of the physical examination, which medical students are routinely taught to this day [2-5]. Audible noise is thought to be produced when a gas and liquid mix during peristalsis [2,6]. There are varying teaching methods for medical students on how to properly auscultate bowel sounds. They range from listening over several areas of the abdomen to one area that radiates over the whole wall to each quadrant prior to percussion [3-5]. Currently, no universal standard for auscultation of the abdomen in the physical examination appears to exist.

Previous research on bowel sounds utilized a variety of invasive methods to try to understand the significance of these noises and yielded limited results [7-9]. Other studies that utilized non-invasive techniques, such as phonoenterography, focused on the pattern and variability of the noises, demonstrating that bowel sounds have multiple different patterns and frequency, postulating this could relate to intestinal motility [10-12]. Now with the common use of ultrasonography, physicians are able to visualize the internal mechanisms of a patient in real-time without radiation or invasive techniques. Applications of ultrasound in abdominal examinations include assessing for peritoneal free fluid, gallstones, abdominal aortic aneurysm, appendicitis, intussusception, and many more [13-16]. Visualization of peristaltic movement with point-of-care ultrasound (POCUS) has been demonstrated to have diagnostic accuracy in the diagnosis of small bowel obstruction [17]. Using ultrasound to visualize peristalsis now presents the new opportunity to further characterize bowel sounds.

The purpose of this study was to determine the relationship between auscultation of bowel sounds with a stethoscope and visualization of peristalsis with an ultrasound. We hypothesized that bowel sounds and peristalsis are not correlated nor compartmentalized.

This article was previously presented as an oral presentation at the 2020 World Congress of Ultrasound in Medical Education on September 14, 2019.

## Materials and methods

This study was designed and approved by the Institutional Review Board, and conducted in accordance with all applicable guidelines and protocols.

Study design

This is a cross-sectional observational study that occurred at a county tertiary care hospital in the United States. The recruitment period was from July to August 2019 through email notification. Volunteers were not acutely ill and had normal anatomy. Participants were screened for eligibility, provided verbal consent, and did not receive compensation for their involvement. Healthy adults with no history of abdominal surgery or trauma and the ability to lay still for fifteen minutes were included. Pregnant patients were excluded. No study follow-up occurred. For consistent data collection, study coordinators utilized a Philips Sparq ultrasound and an Eko Digital Stethoscope (Eko Devices, Inc., Oakland, CA). Bowel sounds were not digitally amplified but were stored using the EKO software app (Eko Devices, Inc., Oakland, CA) for later review and confirmation.

Data measurement

**Figure 1:**
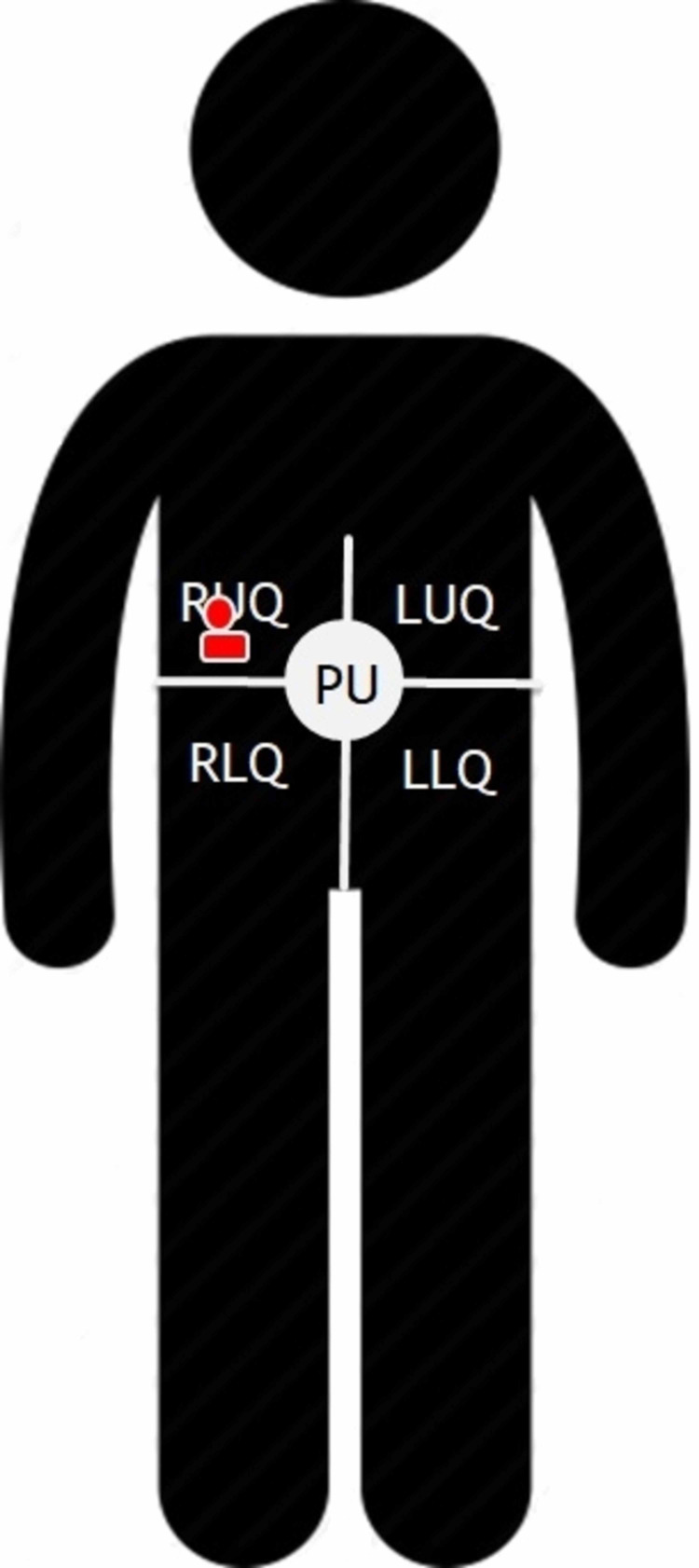
Positioning of stethoscope and ultrasound probe The abdomen was divided into five regions: right upper quadrant (RUQ), right lower quadrant (RLQ), left upper quadrant (LUQ), left lower quadrant (LLQ), periumbilical region (PUR).

For anatomic compartmentalization, the abdomen was divided into five regions, including four quadrants and the periumbilical region (PUR; Figure 1). The four quadrants consisted of the right upper quadrant (RUQ), left upper quadrant (LUQ), right lower quadrant (RLQ), and left lower quadrant (LLQ). Study participants were required to lay quietly in the supine position on a hospital bed for the duration of the study. Each abdomen region was investigated for a time interval of two minutes with continuous monitoring. The ultrasound investigator positioned a curvilinear (C5-1) ultrasound probe on the first region of the abdomen to visualize the lumen of the small intestine. The ultrasound investigator was trained to observe peristalsis of the small intestine. The auscultation investigator placed a stethoscope directly adjacent to the probe on the first region of the abdomen to auscultate for bowel sounds. The recording investigator acted as the event recorder. Throughout the time interval, the ultrasound investigator and auscultation investigator would indicate to the recording investigator when they visualized peristalsis or heard a bowel sound, respectively. Each indication was recorded in real-time by the recording investigator logging the event into one of three categories: solo auscultation of bowel sound (A+), solo ultrasound visualization of peristalsis (U+), or combined auscultation and ultrasound visualization (C+). The number of events of A+, U+, and C+ was recorded from each of the five abdominal regions, for 10 minutes observed overall.

Participant demographic data were not collected. Statistical analyses of each region included Fisher's exact test to determine whether there were significant differences between the number of singleton events (either solo auscultation or solo visualization) and the number of combined events (A+/U+). Significance was determined with p < 0.05. All statistics were performed on STATA version 14.0 (College Station, TX: StataCorp LP).

## Results

A total of 16 participants were evaluated in this study, resulting in the observation of 973 discrete bowel events. There were 202 solo auscultation events, 395 solo visualization events, and 377 combined events. No significant difference in the number of singleton events versus the number of combined events (p < 0.05) was observed.

**Figure 2:**
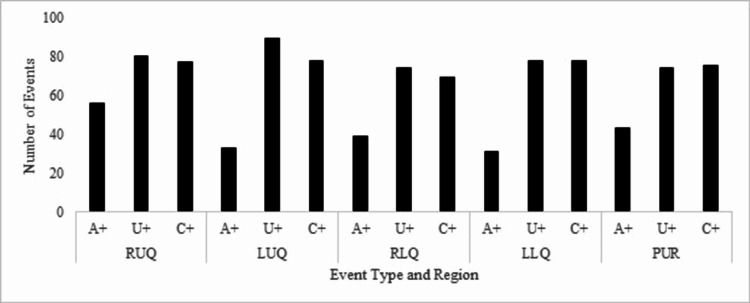
Total number of recorded events per region. Frequency of solo auscultation events (A+), visualization events (U+), and combined events (C+). No significant difference between singleton events compared to combined events (p > 0.05). Right upper quadrant (RUQ), right lower quadrant (RLQ), left upper quadrant (LUQ), left lower quadrant (LLQ), periumbilical region (PUR)

Events were analyzed according to their specific region, as shown in Figure 2, comparing the frequency of singleton events to combined events. There was no significant correlation (p-value range 0.052-1.00) between singleton and combined events in any of the tested regions. A full breakdown of the analysis can be seen in Table 1.

**Table 1:** Statistical analysis of recorded events. Fisher's exact test statistic comparing isolated auscultation (A+) and sonographic (U+) events to combined events (C+) in the five investigated regions. No significant correlation was found. Right upper quadrant (RUQ), right lower quadrant (RLQ), left upper quadrant (LUQ), left lower quadrant (LLQ), periumbilical region (PUR)

Parameter	Fisher’s exact test statistic (p<0.05)
RUQ_A+ to RUQ_C+	1.000
RUQ_U+ to RUQ_C+	0.170
LUQ_A+ to LUQ_C+	0.923
LUQ_U+ to LUQ_C+	0.543
RLQ_A+ to RLQ_C+	0.318
RLQ_U+ to RLQ_C+	0.671
LLQ_A+ to LLQ_C+	0.628
LLQ_U+ to LLQ_C+	0.052
PUR_U+ to PUR_C+	1.000
PUR_A+ to PUR_C+	0.591
Total_A+ to Total_C+	0.130
Total_U+ to Total_C+	1.000

## Discussion

The stethoscope has been one of the physicians' most powerful and most utilized tools since its introduction in the mid-19th century [1]. Its auscultatory power allowed physicians a window into the body. Auscultation of bowel sounds, in particular, has been a mainstay of physical diagnosis for well over a century, with physicians noting its value as early as 1905 [2]. However, recent research on the production and utility of these sounds is relatively sparse, with most studies involving invasive or ex-vivo studies [7-9]. Phonoenterography studies utilized a non-invasive methodology but focused more on characteristics and variability of sounds, rather than the origin of the sounds [10-12]. Ultrasonography has advanced significantly in the last several decades, allowing researchers and physicians a new methodology for observing in-vivo processes. This study investigated direct gut observation with ultrasonography with auscultation, allowing for the investigation of auscultated sounds. 

This study showed no significant difference (p < 0.05) in the number of singleton events versus combined events. This indicates that there was just as likely to be an auscultated bowel sound with no evidence of nearby peristalsis as there was a combined event. Similarly, there were many instances of observed peristalsis with no corresponding bowel sound auscultated in the region. This indicates that while bowel sounds are heard in all quadrants, it does not mean that peristalsis is occurring in that quadrant simultaneously, and there are times that peristalsis occurs in which no bowel sounds are produced.

As discussed previously, there is no true standard throughout physical diagnosis textbooks on the proper way to auscultate for bowel sounds [3-5]. Multiple physical diagnosis textbooks recommend listening to the abdomen in multiple areas for several minutes to thoroughly investigate the bowel sounds [3,4]. Given our study results, the method of listening to bowel sounds in multiple quadrants likely yields little unique information compared to listening in one central region. This is congruent with the physiology proposed by McGee, in which generated sounds radiate across the abdominal wall [5]. This calls into question teaching the methods of auscultation that involve multiple points on the abdomen and the benefit it provides during the examination. 

Further confounding the picture are the many instances of observed peristalsis without an auscultated bowel sound. Many previous articles reference bowel sounds like a way to assess GI motility in patients. However, if there is peristalsis occurring with the absence of auscultated noises, it is unclear exactly how much gut function can be truly gleaned from the presence or absence of bowel sounds. Recent articles have argued for the usefulness of bowel sounds for abdominal pathologies such as mechanical ileus and diffuse peritonitis, yet our results suggest that peristalsis may still be occurring despite a quiet abdomen [18].

In addition to a medical education perspective, the utility of bowel sound auscultation in a clinical setting must be considered. Historically, abdominal auscultation was used to corroborate diagnoses, such as small bowel obstruction. However, more recent articles have shown a sensitivity as low as 0.42 and a specificity of 0.72 for bowel sounds in the setting of small bowel obstruction [19]. Further prospective research noted no usefulness in bowel sound evaluation in diagnoses, with investigators frequently arriving at the wrong diagnosis [20]. There has even been growing talk in the surgical community about whether it is time to abandon abdominal auscultation altogether [21]. Elhardello and Macfie conclude that bowel sounds serve no purpose as a means of assessing gut function. However, they concede that it still serves to ensure close observation of the abdomen and alert clinicians to ileus when there is an overall lack of bowel sounds [21]. 

Limitations

Ultrasound probes have a limited footprint, and thus it is not possible to visualize an entire quadrant of the abdomen at once. Attempts were made to increase visualization by rotating the transducer from sagittal to transverse while sweeping in the quadrant. Given this, it is possible that peristalsis was occurring in a non-visualized part of the quadrant at any given time. In addition, ultrasound focused on the small bowel, so the stomach and colon were not investigated as potential places of peristalsis and bowel sound origination. Finally, while every attempt was made to place the stethoscope and ultrasound probe directly adjacent, it is impossible for them to capture the same footprint, thus there is a small margin of error in the window of investigation for this study.

## Conclusions

While considered an essential part of the physical exam, bowel sounds have limited use in a targeted manner. This study demonstrates that bowel sounds’ origin is often unrelated to the space in which they are auscultated. This calls into question the practice of educating medical students to listen for bowel sounds in multiple regions of the abdomen when they are likely not contained to the region in which they are heard. It also calls into question the usefulness of bowel sounds in a clinical setting, as peristalsis was often observed in the absence of auscultated sounds, indicating that auscultated bowel sounds are not a good measure of the level of peristalsis occurring in the gut.
